# Nitroglycerin: a comprehensive review in cancer therapy

**DOI:** 10.1038/s41419-023-05838-5

**Published:** 2023-05-12

**Authors:** Mélina Meunier, Aline Yammine, Ali Bettaieb, Stéphanie Plenchette

**Affiliations:** 1 Laboratoire d’Immunologie et Immunothérapie des Cancers (LIIC), EA7269, Université de Bourgogne, Dijon, France; 2 LIIC, EPHE, PSL Research University, Paris, France

**Keywords:** Cancer therapy, Preclinical research, Outcomes research

## Abstract

Nitroglycerin (NTG) is a prodrug that has long been used in clinical practice for the treatment of angina pectoris. The biotransformation of NTG and subsequent release of nitric oxide (NO) is responsible for its vasodilatating property. Because of the remarkable ambivalence of NO in cancer disease, either protumorigenic or antitumorigenic (partly dependent on low or high concentrations), harnessing the therapeutic potential of NTG has gain interest to improve standard therapies in oncology. Cancer therapeutic resistance remains the greatest challenge to overcome in order to improve the management of cancer patients. As a NO releasing agent, NTG has been the subject of several preclinical and clinical studies used in combinatorial anticancer therapy. Here, we provide an overview of the use of NTG in cancer therapy in order to foresee new potential therapeutic avenues.

## Facts

- Nitroglycerin is a vasodilatating agent used to treat or prevent angina.
- Nitroglycerin increases the effects of various cancer treatments in preclinical studies.
- Variable results so far for combination therapy with nitroglycerin in clinical trials.

## Open questions

- What is the role of NTG-mediated ERP effect in the antitumor response of nitroglycerin?
- Which NTG-based combination therapy would more appropriate to improve the outcome of patients?
- Can NTG may regulate other TNF receptors via NO-dependent post-translational modifications?
- Can tumor microenvironment components be utilized as biomarkers to predict the clinical benefit of nitroglycerin in combinatorial therapies?

## Introduction

Nitroglycerin (NTG), also called glyceryl trinitrate (GTN), is an organic nitrate molecule that has entered the medicinal world for more than 170 years. Just like its chemical properties, the scientific history of NTG is remarkable and astonishing.

NTG was synthetized by the Italian chemist Ascanio Sobrero in 1847 via the nitration of glycerol using a mixture of nitric and sulfuric acids causing a highly explosive reaction unless the mixture is cooled. Some years later, Alfred Nobel patented and manufactured dynamite, a stabilized form of NTG. In the late 1860s, some factory workers in explosives industry, suffering from angina pectoris or heart failure, reported a significant relief of pain only on workdays thus attributed to NTG exposure. In the meantime, the development of homeopathic medicine (based on the principle of cure by the similitude “like cures like”) was the cornerstone for the entry of NTG in the *Pharmacopoeia* and Medicine. In parallel, Brunton discovered the ability of a nitric-containing compound, amyl nitrite, to lower blood pressure and relieve anginal pain [1, 2]. Based on all these observations, William Murrel successfully treated angina pectoris for the first time with NTG in 1878. NTG-induced smooth muscle relaxation was further established [3] and still remains to date the milestone therapy for the relief of anginal pain. The molecular mechanism involved was then attributed to nitric oxyde (NO). Ferid Murad, Robert Furchgott and Louis Ignarro were awarded the 1998 Nobel Prize in Physiology/Medicine for their discoveries concerning NO as a signaling molecule in the cardiovascular system [4].

NO-released by NTG is known as a direct activator of soluble guanylyl cyclase (sGC), which activates the production of guanosine monophosphate cyclic (GMPc) and ultimately results in a signaling pathway that causes vascular smooth muscle relaxation [5]. The biological properties of NO have led to the search for new therapeutic uses of NTG. For several decades, numerous randomized clinical trials examined the effectiveness of NTG in a wide spectrum of human disorders including anal fissures [6–8] tendinopathies [9], cervical ripening [10], the prevention of mastectomy skin flap necrosis (MSFN) [11], and the prevention of acute pancreatitis post-endoscopic retrograde choliangiopancreatography (ERCP) [12–14].

Since the entry of NTG in the medicine, the understanding of the molecular mechanism of NTG bioactivation and the biological action of NO or NO-containing metabolite continues to intrigue and fuel research for novel potential therapeutic avenues.

Either produced by NO synthases or delivered by NO donors, NO plays a role in a broad spectrum of physiological and pathophysiological processes. NO exists as a free radical gaseous molecule (•NO) highly reactive on molecular oxygen (O_2_), iron, superoxide anion (O_2_•^−^) characterized with the production of subsequent reactive nitrogen species, nitrogen dioxide (•NO_2_) then nitrite (NO_2_^−^), nitrate (NO_3_^−^) and peroxynitrite (ONOO^-^) respectively [15]. Important NO biological effects (•NO or NO-derived from RNS) are mediated by protein post-translation modifications such as metal-nitrosylation, the reaction of NO with a transition metal (e.g. heme group), nitrosation the NO-dependent covalent modification of a tyrosine residue (i.e. hydroxyl group) and S-nitrosylation the NO-dependent non-covalent modification of a cysteine residue (i.e. thiol group) [16].

The biological outcome of NO has been shown to be cytoprotective or cytotoxic depending on various factors including concentration, subcellular location, the chemical redox state of the cellular microenvironment and time of NO exposure [17]. The dichotomous effects of NO have been well established in tumor cells. High concentration of NO (of the order of µM) has a cytotoxic/antitumoral effect whereas low concentration of NO (of the order of nM) has a cytoprotective/carcinogenic effect [17, 18]. Increase in the concentration of NO using NO donors represent an interesting novel therapeutic strategy. Many preclinical and clinical studies have examined the therapeutic potential of NTG to treat different types of cancer yet. Whether NTG may provide a clinical benefit for cancer patients have been investigated for several decades and is still a matter of clinical research. In this review, we aim to provide an updated insight on the use of NTG in cancer therapy.

## Pharmacokinetic of NTG

### NTG formulations

NTG exists under various formulations for oral and sublingual use (tablets and sprays), transdermal use (ointments and patches), and intravenous use (liquid) [19]. The routes of administration of NTG have a significant impact on its biotransformation and pharmacokinetic. When administered orally, NTG undergoes an exhaustive first-pass hepatic extraction with a plasma concentration frequently below the detection threshold (~0.1 ng/ml) [20, 21]. Sublingual administration of NTG has a short-lived effect and prevents the first-pass hepatic metabolism which results in greater NTG plasma concentrations (concentrations reached within 2–5 min with 0.5 mg NTG sublingual tablet: 1.4 ± 0.1 ng/ml [22]; 0.8 mg NTG sublingual spray: 3.96 ng/ml)) [23]. Transdermal administration of NTG provide a longer-lasting effect [24]. It also offers the advantage of avoiding first-pass hepatic extraction and constitutes a controlled-delivery system with steady plasma concentration even though the patch is replaced [25]. When given intravenously (like sublingually) NTG has a short plasma half-life of 2–3 min [24, 26].

### Nitrate tolerance

The attenuation or full loss of the NO-dependent hemodynamic and anti-ischemic effects of NTG, often known as “nitrate tolerance,” severely restricts the therapeutic effectiveness of continuous NTG administration regardless the route of administration [27]. Vascular tolerance is thought to be multifactorial due to the depletion of intracellular thiols, inhibition of nitrate bioactivation enzymes, decreased NO bioavailability caused by changes in eNOS expression and activity and/or increased phosphodiesterase (PDE) activity, and the reduction in cGMP-dependent protein kinase (cGK) activity [28, 29].

### Adverse effects

The systemic toxicity of NTG is minimal. Headache is the most frequent negative effect of nitrate therapy. Dizziness and syncope are less frequently reported because nitrate-induced hypotension frequently rises heart rate, which offsets a decline in stroke volume. Flushing, palpitations, and hypotension with reflex tachycardia are the most frequent cardiovascular side effects linked to NTG [30].

### Bioactivation of NTG

#### NTG metabolites

NTG biotransformation was first defined by Bennett et al. as two mechanisms. The first one is a mechanism-based biotransformation (or bioactivation) in which NTG is denitrated and transformed into the vasoactive compound NO that activates the soluble guanylate cyclase, which subsequently upregulates intracellular cyclic guanosine 3′,5′- monophosphate (cGMP) and causing muscles to relax. NO generation is coupled with the production of primarily of 1,2-glyceryl dinitrate (1,2-GDN) and a considerably lower amount of 1,3-glyceryl dinitrate (1,3-GDN). The second mechanism is a clearance-based biotransformation (or metabolic clearance) in which NTG is metabolized into 1,3-GDN and a nitrite (NO_2_^−^) with poor vasodilatory capabilities [31].

Regardless of the method of administration, NTG metabolism generates dinitrate metabolites 1, 2- and 1, 3-GDN, which in turn generate 1- and 2-glyceryl mononitrate (1- and 2-GMN). When compared to NTG, the GDNs and GMNs plasma concentrations are often 10 times and 100 times higher respectively [26]. The metabolites have substantially longer half-lives than NTG with 30–60 min for GDNs and 5–6 h for GMNs, which explains their higher plasma concentration [26]. It was found that 1, 2-GDN plasma levels are always greater than 1, 3-GDN levels, and that 2-GMN concentrations are always higher than 1-GMN concentrations. When administered by a transdermally route, 1,2-GDN was shown to be typically 4 times more concentrated than 1,3-GDN and 2-GMN was ~6 times more concentrated than 1-GMN [32]. Following sublingual (0.4 mg) and intravenous administration (10, 20, and 40 μg/min) of NTG, the concentration of 1, 2-GDN may be 4.6–7.4 times greater than that of 1, 3-GDN, respectively [33].

#### Mechanisms of NTG bioactivation

**Fig. 1:**
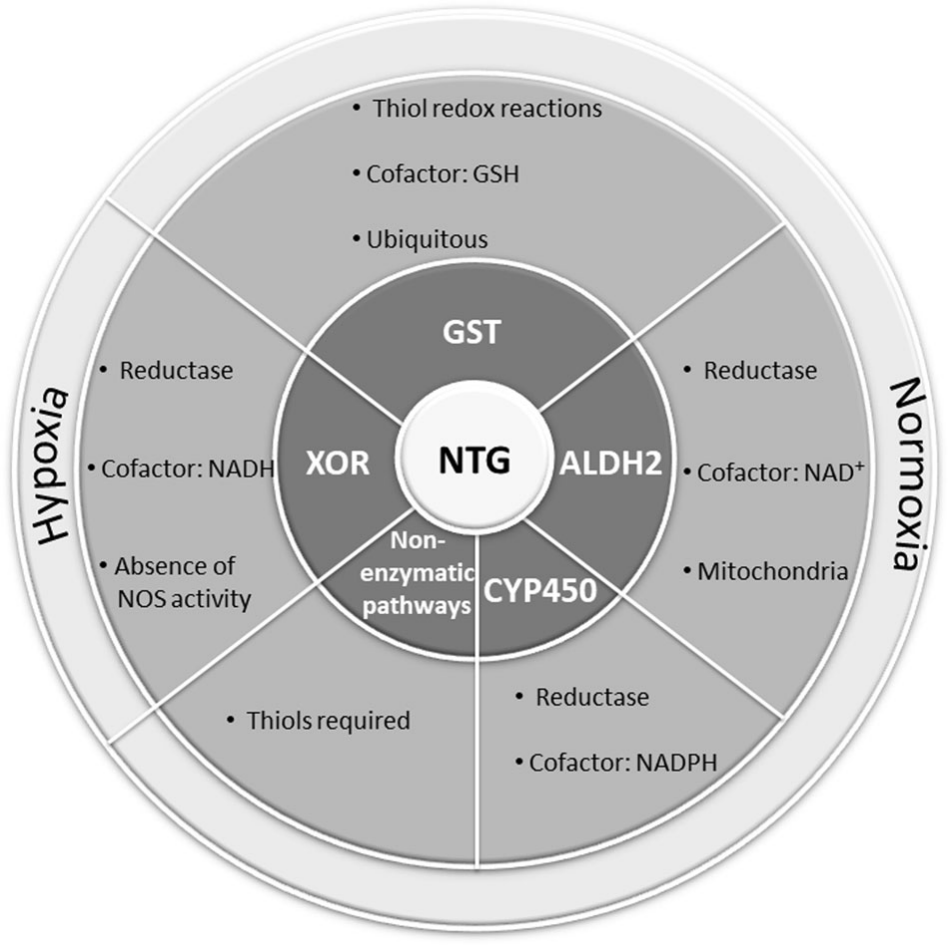
Bioactivation of NTG. NTG bioactivation generates (at different quantities and ratios) 1,2-GDN, 1,3- GDN, inorganic nitrite, and NO (or -SNO). The mechanisms responsible for this transformation include enzymatic and non-enzymatic pathways. The activation of the non-enzymatic pathways requires the presence of thiol or reduced sulfhydryl compounds; however, it is most likely compared to clearance-based biotransformation [26]. The enzymatic pathways are mainly mediated by four enzymes: the ubiquitous glutathione S-transferase (GST) which converts NTG into 1,2-GDN, 1,3- GDN leading to relaxation of vascular smooth muscle [36], the hepatic cytochrome P450 (CYP450) which promotes predominantly the generation of the vasoactive NO [35] and other CYP isoenzymes such as CYP 1A2, 2A6, 2C9, 2E1, 3A4 and 2J2 with roles in NTG biotransformation has not been fully studied [99], the mitochondrial Aldehyde Dehydrogenase 2 (ALDH2) which mainly catalyzes the generation of 1,2-GDN and nitrite [100] and the endothelial XOR that converts NTG into NO using NADH as a cofactor, under hypoxic conditions. XOR has a complementary function to NOS activity rendered inactive in absence of molecular oxygen [37].

For many years, the process by which NTG undergoes biotransformation to release NO and its dinitrate metabolites remains not fully understood. Hence, numerous NTG biotransforming enzyme-dependent and independent mechanisms have been proposed (Fig. 1) [34]. Four enzymes, including cytochrome P450 (CYP450), glutathione-S-transferase (GST), xanthine oxidoreductase (XOR), and aldehyde dehydrogenase (ALDH), have been associated with the biotransformation of NTG [35–38].

ALDH2 is a key NTG bioactivator and it is also involved in detoxification of ethanol. It exhibits many enzymatic activities including dehydrogenase, esterase and denitration. Of note, an inactive mutant of ALDH2 (Glu504Lys) is found in about 40% of the East Asian population which is responsible for defective alcohol metabolism. The vascular response to NTG metabolism investigated in ALDH2 Glu504Lys versus ALDH2 wild-type individuals has shown contradictory clinical results: either no effects [39] or a lack of an efficacious response [40] associated to ALDH2 Glu504Lys. This suggests the existence of alternative pathways for NTG biotransformation. Furthermore, site directed mutagenesis of ALDH2 suggests three distinct pathways of NTG biotransformation that involve different active site residues (Cys302, Cys301/303, Glu268) [41]. Although the mechanism for ALDH2-catalysed NTG denitration is not fully understood, at least Cys302 and Glu268 appear essential in this process reported for vascular bioactivation of NTG [42, 43]. ALDH2 polymorphisms (Glu504Lys) is also correlated with occurrence and progression of cancer but the role is only partially understood [44]. Altogether, given the existence of alternative pathways, it is much likely that the influence of ALDH2 polymorphisms on NTG biotransformation in cancer cells may be rather limited.

Polymorphisms exist within GST and CYP450 genes which may affect their enzyme catalytic activity. For example, GSTM1, GSTT1-null genotypes and GSTP1 rs1695 polymorphism have been correlated as an increased risk factor for a variety of cancer type [45, 46].

We can still speculate that mutation/defective expression of wild type gene or upregulation of a defective catalytic variant in cancer of the enzyme responsible of NTG biotransformation may affect NO delivery and post-translational modification. Further investigations are required to clearly define this point.

## Preclinical studies for the combination of antitumor therapies and nitroglycerin

### Effect of nitroglycerin on the tumor microenvironment

#### Nitroglycerin and Cytokine signaling

##### Tumor necrosis factor (TNF)/TNF receptors superfamily members

Tumor necrosis factor (TNF) and TNF receptors superfamily members play a role in the pathogenesis of various diseases including cancer. The TNF ligands consist of 19 members, well-documented for their engagement in signaling pathways resulting in inflammation, proliferation, cell death, migration/invasion, angiogenesis, and metastasis [47]. In particular, TNFα/TNF-R1, FasL/Fas and TRAIL/DR4 or DR5 systems exert pleiotropic effects, associated with both tumor-promoting and tumor-suppressing effects in the tumor microenvironment (TME). TNF ligand/TNF receptor trimeric assembly and secondary formation of clusters (for category II TNF receptors such as transmembrane Fas, DR4 and DR5) is required for the full activation of the system [48]. NTG has been shown to significantly enhance FasL-mediated cell death by apoptosis in mammary and colon cancer cell lines [49]. S-nitrosylation of Fas in its cytoplasmic part (S-nitrosylation of cysteine residues 199 and 304) was found associated with this process. Particularly, S-nitrosylation at cysteine 304 promotes redistribution of Fas to lipid rafts, formation of the death-inducing signal complex and induction of cell death by apoptosis [49].

An increase in understanding the mode of action of NTG in tumor-suppressive effects brings to the fore a NO-dependent regulation of the TNF ligands/TNF receptors superfamily signaling pathways [50]. In mammary and colon cancer cells (from human and murine origin), NTG induces a switch of the TNFα/TNF-R1 signaling pathway from the classical survival NF-ƙB pathway to a pro-apoptotic cell death pathway [51]. The switch occurs in a NO-dependent molecular mechanism via the S-nitrosylation and inactivation of the cIAP1 E3 ligase activity (cysteine at position 571). In absence of K63 ubiquitinated RIP1, the TNFα/TNF-R1 system NTG mediates a cell death signaling pathway involving the formation of a complex II [51]. The E3 ligases cIAP1/2 are long-time active targets for drug development. Smac-Mimetic (SM) compounds have been extensively studied and developed to counteract cIAPs by inducing their autoubiquitination and proteasomal degradation [52]. As SM, NTG induces TNFα-dependent cancer cell death by apoptosis due to the loss of cIAP1 E3 ligase activity by a distinct molecular mechanism.

To date no study has explored whether NTG might display a cellular function via other TNF receptors. Only one study reported the S-nitrosylation of DR4 (not for DR5), specifically by the NO donor nitrosylcobalamin and its role in sensitizing ovarian cancer cell lines to TRAIL-induced apoptosis [53].

##### IL-6-dependent migration/invasion

Interleukin-6 (IL-6) is a critical cytokine and key inflammatory mediator within the TME that is strongly implicated in almost all hallmarks of cancer such as inflammation, cell proliferation/inhibition of cell death, migration/invasiveness, and metastasis formation [54]. The consideration of IL-6 levels detected in the serum of various type of cancer patients as potential diagnostic and predictive biomarker has been discussed in the literature [55–57]. Whether NTG may modulate the level of inflammatory mediators in blood has been investigated. Interestingly, continuous therapy with transdermal NTG (0.6 mg/h, 24 h/day for 7 days) or no therapy in healthy volunteers was not associated with changes in IL-6, TNFα and other vascular inflammation biomarkers [58]. While NTG therapy does not appear to have an impact on the level of IL-6 in human serum, it does affect IL-6 signaling. The IL6/JAK/STAT3 signaling pathway is frequently activated in various human cancers driving cell proliferation, migration/invasion, and cancer metastatic dissemination. As one of the most dysregulated signaling pathway in cancer, especially in breast cancer, many therapies targeting the IL6/JAK/STAT3 signaling pathway have been developed and studied [59]. More recently, it has been demonstrated that NTG has an inhibitory effect on the IL6/JAK/STAT3 signaling and consequently can prevent the migration and invasion of triple-negative breast cancer (TNBC) cells examined in vitro. Mechanistically, NTG mediates the S-nitrosylation of JAK2 which inhibits the phosphorylation of JAK2 and subsequent activation to phosphorylate its downstream substrate STAT3 [60].

#### Nitroglycerin and hypoxia-mediated immune escape

**Fig. 2:**
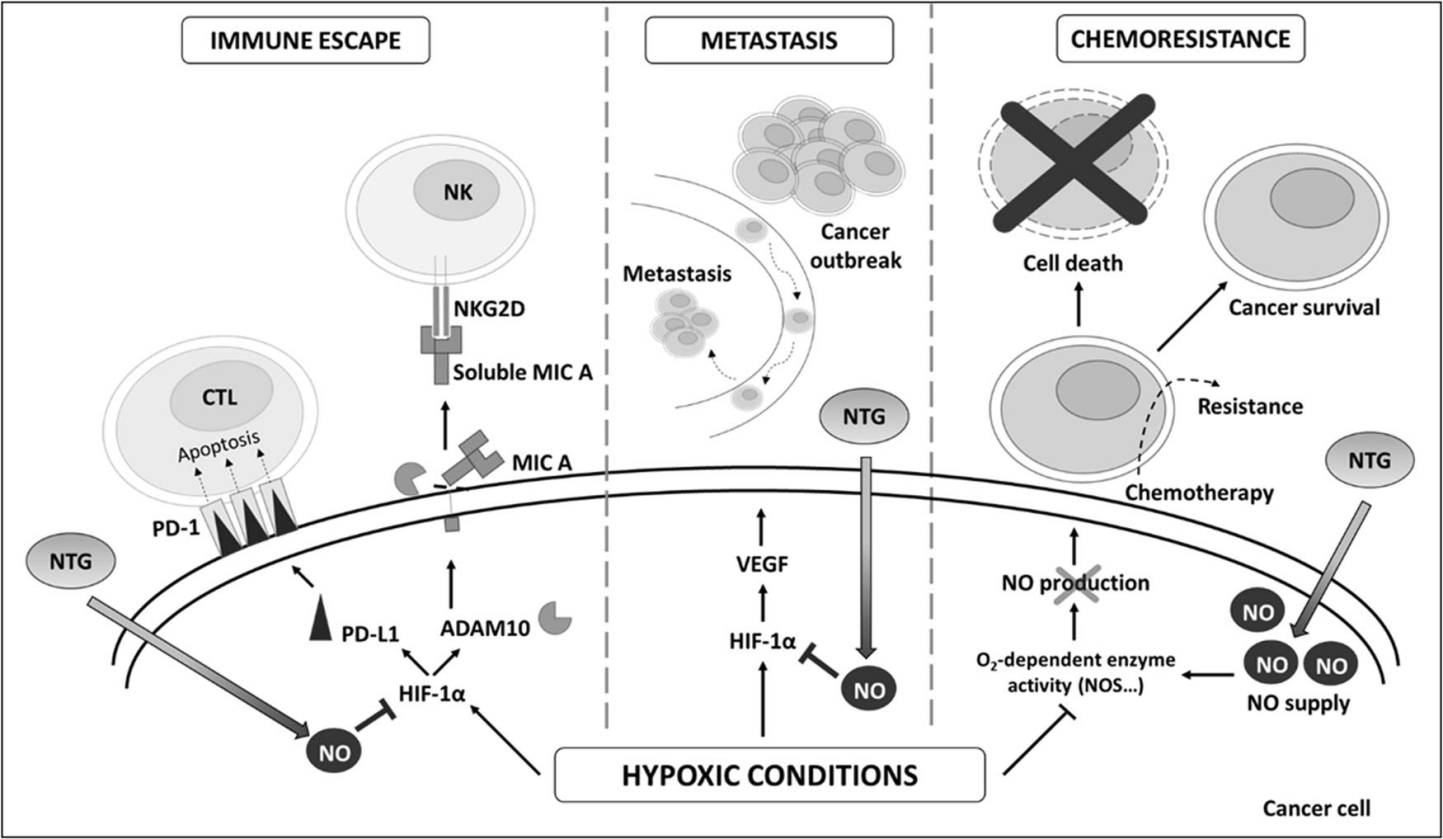
Effect of NTG under hypoxia on cancer cells. In hypoxic conditions, HIF-1α contributes to the escape from innate and adaptative immune surveillance, the formation of metastases and resistance to chemotherapies. HIF-1α (1) upregulates ADAM10 inducing the shedding of MICA at the surface of cancer cell and the resistance to killing by immune cells (NK, LAK and T cells) [64]; (2) upregulates PD-L1 which interacts with PD-1 on cytotoxic T lymphocytes and leads to inhibition of the adaptive immune response [65]; (3) upregulates VEGF which promotes angiogenesis and the formation of metastases [101]. Hypoxia inhibits O_2_-dependent enzyme activity which decreases the endogenous NO production and promotes cancer cell resistance to chemotherapy and cell survival. NTG, via its intracellular metabolism and NO release, inhibits hypoxia-mediated HIF-1α accumulation and counteracts these processes.

Hypoxia contributes to the escape of both innate and adaptative immunity which contributes to malignant progression. There is a growing body of evidence that suggests that NO donors, such as NTG, could immunosensitize tumor cells (Fig. 2).

The expression of MHC class I chain-related molecules (MICA and MICB) plays an important role in tumor surveillance by natural killer (NK) cells, lymphokine-activated killer (LAK) cells and cytotoxic T (T) cells [61]. Hypoxia-mediated shedding of MICA at the surface of human DU-145 prostate cancer cells, and subsequent resistance to killing by immune cells, is linked to an impaired NO signaling. In hypoxic conditions (0.5 % O_2_), low concentration of NTG (10 nM) significantly immunosensitize DU-145 cancer cells, most likely by restoring NO/cGMP signaling [62, 63]. Mechanistically, NTG interferes with the hypoxia-induced accumulation of ADAM10 (a disintegrin and metalloproteinase domain-containing protein 10), a gene target of the transcription factor HIF-1α (Hypoxia-inducible factor 1-alpha) that encodes for a metalloproteinase required for the shedding of MICA (Fig. 2). An antitumoral and immunotherapeutic effect of NTG (continuous administration of patch, 1.8 µg/h) further support the finding [64]. In addition, hypoxia-mediated up-regulation of PD-L1 (programmed death-ligand 1), evidenced in human breast and prostate cancer cells, and in murine melanoma and mammary carcinoma cells, leads to resistance to CTL (cytotoxic T lymphocyte)-mediated lysis (Fig. 2). Low dose of NTG counteracts this effect and sensitizes tumor cells to CTL-mediated lysis [65].

#### Nitroglycerin and angiogenesis

Peripheral circulation of bone marrow-derived stem cell population, endothelial progenitor cells (EPCs), plays a critical role in sustaining angiogenesis by their differentiation into endothelial cells and secretion of proangiogenic factors [66]. The impact of continuous exposure of various doses of NTG on human peripheral blood-derived EPCs was investigated. Transdermal administration of NTG (0.6 mg/h) given continuously for 7 days to healthy volunteer significantly increased the percentage of circulating cells expressing the EPC marker [67]. Ex vivo, exposure of NTG dose range (100–1000 nM) to human peripheral blood-derived EPCs (isolated from healthy individuals) was associated with increased EPCs apoptosis in a NTG-dose-dependently manner [67]. Ex vivo, continuous exposure of moderate concentrations of NTG (≤7.5 mg/l) increases the proliferative capacity of EPCs (isolated from patients with coronary artery disease) whereas exposure of higher concentration of NTG (≥15 mg/l) inhibits the proliferation of EPCs [68].

Interestingly, the adverse effect of continuous exposure of high doses of NTG on endothelial dysfunction represents an opportunity in cancer therapy to disrupt tumor angiogenesis. From a mechanistic point of view, it has been demonstrated that NTG-induced endothelial dysfunction may be due to phosphorylation and S-glutathionylation of eNOS [69]. In support to these studies, long-term exposure of NTG is linked to Akt S-nitrosylation and inactivation in endothelial cells and further impairment of angiogenesis [70].

#### Nitroglycerin and metastasis

Some insights indicate that NTG may contribute to counteract the shaping of metastasis (Fig. 2). The anti-metastatic effects of NTG have been examined on aggressive subtypes of metastatic breast and melanoma cancers. Basal-like TNBC is the most aggressive subtype of breast cancer associated with a poor prognosis, invasiveness, and distant metastasis [71, 72]. Recently, it was shown that in vivo metastatic potential of 4T1 murine TNBC cells to form lung nodules was delay following NTG therapy [60]. In that study, in vitro migration and invasion assays carried out with both 4T1 murine and MDA-MB-31 human TNBC cells were significantly inhibited by NTG treatment (250 µM) [60]. A previous study has shown that experimentally induced hypoxia (0.5% O_2_) increased the in vitro invasiveness of MDA-MB-231 cells which was prevented with low concentration of NTG (1 pM and 0.1 µM) [73]. In support of this finding, the anti-metastatic effect of low concentration of NTG (20 pM) was observed in vivo in hypoxia-mediated lung nodule formation using B16F10 murine metastatic melanoma cells. The experimental metastasis assay was established using C57Bl/6 mice injected intravenously with B16F10 cells that were pre-incubated for 12 h in hypoxic (1% O_2_) versus normoxic (20% O_2_) conditions, and treated with or without NTG [74].

### Nitroglycerin as a sensitizing agent in cancer therapies

#### Chemosensitization

The chemosensitizing ability of NO has been demonstrated in different cellular contexts. Tumor hypoxia is known to increase resistance to chemotherapeutic agents and also radiotherapy (Fig. 2). One of the mechanisms associated with hypoxia-induced drug resistance is the inhibition of endogenous NO production (molecular O_2_ being required for NOS activity) also referred as “hyponitroxia” [75]. This finding supported the rationale for the use of NO donor to compensate for the loss of NO production in order to alleviate chemoresistance acquired in tumor hypoxia. In vitro studies have demonstrated that low concentrations of NTG (0.1 nM to 1 µM) significantly reduce hypoxia-induced chemoresistance of MDA-MB-231 TNBC cell line to doxorubicin [75] and to paclitaxel [76]; B16F10 melanoma cell line to doxorubicin [75]; DU-145 and PC-3 prostate cancer cell lines to doxorubicin [77] and to paclitaxel [76]. The chemosensitizing effect of NTG (by continuous transdermal delivery) to doxorubicin was also evidenced in vivo using a xenograft mouse model of human prostate cancer cell. Multicellular resistance, observed when cancer cells grown in spheroids, to doxorubicin is attenuated in MDA-MB-231 spheroids treated by NTG [78].

Further studies reported by the same team indicated that NTG induces the chemosensitization of hypoxic tumor cells in a cGMP-dependent signaling manner in both monolayer culture and spheroids [76, 78]. Acquired resistance to docetaxel is frequently observed in metastatic castration resistant prostate cancer (mCRPC). Interestingly, established docetaxel-resistant mCRPC cell lines (DU145-DR and PC3-D12) were found more sensitive to NTG-induced cytotoxicity than the parental cell line (DU-145 and PC-3) [79].

In addition, NTG was shown to enhance cisplatin-induced cytotoxicity in human NSCLC cell lines A549 and H1703 in vitro [80]. In human colon carcinoma cells SW480, cytotoxity-induced by M1 macrophage-derived conditioned medium pre-treated by the standard chemotherapies 5-fluorouracil/oxaliplatin is significantly enhanced by NTG. TNFα-induced by 5-fluorouracil/oxaliplatin-induced is associated with the enhanced cytotoxicity mediated by NTG [51]. The activity of NTG on the TNF signaling pathways has been described above (section Tumor necrosis factor (TNF)/TNF receptors superfamily members).

#### Photo-sensitization

One preclinical study supports the potential benefit of combining photodynamic therapy (PDT) and NTG on human retinoblastoma tumors xenografted subcutaneaously on mice [81]. A low dose of NTG ointment was applied on the skin of tumor-bearing animals one hour before the PDT treatment (two photosensitizing agent injection). In mice treated with NTG, a significant increase in light intensity detected by Magnetic resonance imaging (MRI) in tumor tissue was observed after PDT compared to the control group (without NTG treatment). The combination of PDT with NTG decreased the tumor volume below its initial value [81].

#### Sensitization toward other molecules

The combination of NTG with a nonspecific kinase inhibitor, H89, synergistically induces apoptosis in colon cancer cells. Such effect is dependent of ROS production and protein kinase G activation and also the P2-purinergic receptors P2X3, P2Y1, and P2Y6 [82].

In another study, when combined with the valproic acid (an inhibitor of histone deacetylase) NTG decreases cell viability and induces apoptosis of human leukemia cells [83].

More recently, it has been reported that NTG can synergistically enhance the cytotoxicity and cell growth inhibition triggered by the anti-cancer drug pemetrexed (an anti-folate) in non-small-cell lung cancer (NSCLC) cells. This effect is associated with Akt and ERK1/2 inactivation and radiation-sensitive 52 (Rad52) (playing a crucial role in DNA repair) downregulation [84]. The antitumor effect of pemetrexed was shown to be enhanced by NTG in a xenograft model of lung cancer in mice. This synergistic effect was significantly reversed by a selective inhibitor of the NO-sensitive guanylyl cyclase, 1*H*[1,2,4] oxadiazolo[4,3-a]quinoxaline-1-one (ODQ), suggesting that the anticancer effect is dependent on the cGMP signaling pathway [85].

Recently, in vivo investigation of the effect of NTG associated to metformin (an anti-diabetes drug) in hamsters-bearing fibrosarcoma tumors has been conducted. The combination of NTG and metformin significantly inhibited fibrosarcoma cell growth without toxicity, compared to monotherapy or control. This antitumor effect is also associated with the inhibition of tumor vasculature, the increase of apoptosis, the inhibition of glucose metabolism and the inhibition of NO metabolism [86].

## Clinical studies for the combination of antitumor therapies and nitroglycerin

### Non-small cell lung cancer

#### Chemotherapeutic treatment

##### Docetaxel and carboplatin

The first clinical trial to test the anticancer effect of NTG in combined therapy was conducted by Yasuda et al. in 2006 [87]. Seventeen patients with lung adenocarcinoma and stable angina pectoris were treated with NTG patches (25 mg daily) during 3 months before surgery. The patients were all suffering from angina pectoris and likely to receive NTG for the treatment of this pathology. However, the continuous NTG treatment without chemotherapy did not have a clinical benefit in the prolongation of time to progression (TTP) after surgery [87]. During this study, the long-term effects of NTG on different proteins (HIF-1α, P-gp, VEGF, p53, and activated p53) were studied. The treatment with NTG decreases the rates of immunoreactive cells for HIF-1α protein and the level of VEGF (vascular endothelial growth factor) protein was closely associated with HIF-1α and P-gp (P-glycoprotein) protein levels in cancer tissues after operation. These results suggest that NTG-induced reduction of HIF-1α level leads to a reduction in VEGF level and subsequent reduction of angiogenesis.

In parallel, 29 patients with non-operable advanced lung adenocarcinoma were treated with NTG patches (25 mg daily for 5 days between 3 days before and 2 days after each cycle of chemotherapy) associated with docetaxel (on day 1) and carboplatin (on day 1) for four cycles maximum. The authors showed that the NTG may improve chemosensitivity to docetaxel and carboplatin in patients with lung adenocarcinoma. This effect of NTG treatment was strongly associated with the decreasing of the level of VEGF and P-gp via the reduction of HIF-1α [87]. In 2016, He and colleagues have also investigated the efficacy and the safety of the combination of NTG with docetaxel (on day 1) and carboplatin (on day 2) for the treatment of 70 elderly patients (≥65 years) with advanced NSCLC complicated with coronary heart disease (CHD). NTG was administrated via intravenous micro-pump during 24 h in continuous from the start of the chemotherapy. The injection speed was gradually increased from 20 to 50 µg/min and maintained at 50 µg/min. The authors observed a significant increase of tumor response rate (25.00% vs 52.63%), of disease control rate (40.60% vs 65.80%) and a median Overall Survival significantly longer (OS, 10.8 vs 8.3 months) in the treatment group compared with the control group. Moreover, the incidence of angina pectoris and myocardial infarction were significantly lower in the treated group [88]. So, this combination can be considered as a safe and effective option to improve the efficacy of docetaxel and carboplatin and treated NSCLC patients with CHD.

##### Vinorelbine and cisplatin

In 2006, Yasuda et al. conducted a randomized phase II clinical trial to investigate the efficacy and safety of NTG combined with vinorelbine and cisplatin in 120 patients with previously untreated stage IIIB/IV NSCLC [89]. The combination improved the response rate (72% vs 42%), the TTP (327 vs 185 days) and the median survival time (413 vs 289 days) in the NTG compared with the placebo arms, without the appearance of major adverse effects [89]. These results were confirmed by Reinmuth et al. with a study evaluating the effects of NTG combined with oral vinorelbine and cisplatin in 66 Caucasian patients with stage IIIB/IV NSCLC [90]. In this study, the addition of NTG increased numerically but no significatively, the objective response rate (ORR, 35.3% vs 18.8%) and the disease control rate (DCR, 61.8% vs 53.1%) compared to placebo group. However, there were no differences in the median time to progression (TTP) and the median overall survival (OS) between NTG and placebo groups. The results seem to confirm the previous results reported by Yasuda et al. in Asian cohort [89] despite the low sample size.

In 2014, a new phase II clinical trial combining with vinorelbine and cisplatin with concurrent NTG and radiotherapy for treatment of locally advanced NSCLC (35 patients) has been realized. The NTG patch was administrated for 5 days (25 mg 1 day before and 4 days after chemotherapy induction and consolidation) and all-day during chemo-radiotherapy for a total of six cycles of chemotherapy. This study demonstrated that 63% of patients present an overall response (OS) after induction of chemotherapy and 75% an OS after chemo-radiotherapy. Moreover, the improved OS was associated with reduced VEGF levels. Thus, the addition of NTG to chemotherapy and radiotherapy can be used for the treatment of locally advanced NSCLC without toxicity [91].

##### Carboplatin, paclitaxel and bevacizumab

In parallel, a randomized phase II clinical trial combining NTG with carboplatin, paclitaxel, and bevacizumab was realized on 223 chemo-naïve patients with stage IV nonsquamous NSCLC. The patients were randomized to receive four cycles of chemotherapy every 3 weeks with or without NTG patches (15 mg daily for 5 days between 2 days before, the day of, and 2 days after each cycle). Unfortunately, the association of NTG with carboplatin, paclitaxel, and bevacizumab did not increase the response rate (38% vs 54%), the progression-free survival (PFS, 5.1 vs 6.8 months) and the overall survival (OS, 9.4 vs 11.6 months) in patients with stage IV nonsquamous NSCLC treated with NTG patch compared to placebo group [92]. So, the dramatically decrease of the VEGF levels by the bevacizumab could be responsible of the loss of NTG enhancement potential in patients with stage IV nonsquamous NSCLC.

While the clinical trial by Dingemans et al. [92] shows that NTG does not improve treatment efficacy (carboplatin, paclitaxel and bevacizumab), Jong et al. investigated whether a predicted outcome could be based on early response assessment using [18F] FDG PET imaging from available data [93]. The addition of NTG did not reduce FDG uptake. However, this method identified more tumor responders than chemotherapy-based response assessment, but this was not correlated to progression-free survival (PFS) or overall survival (OS). These results could be due to a lower NTG dose than the one used in Yasuda’s study or to the timing of the [18F] FDG PET shortly after the bevacizumab infusion and so to an interference with bevacizumab [93].

The combined effect of NTG with chemotherapies against the NSCLC seems to depend on the type of chemotherapy used. A rigorous, multicenter, phase III clinical trial was completed in 2015, on 372 advanced NSCLC patients treated with one of five prespecified platinum-based doublets as first-line chemotherapy (carboplatin and gemcitabine (79%) or carboplatin and paclitaxel (18%) or vinorelbine and cisplatin (2%)). The patients were treated with NTG patches (25 mg) two days before, the day of, and two days after, each chemotherapy infusion. Chemotherapies were injected every 3 weeks for a maximum of four to six cycles in the absence of progressive disease or prohibitive toxicity. Unfortunately, this study was stopped because, during the first interim analysis (270 patients), the NTG had no demonstrable effect either on progression-free survival (PFS, 5.0 vs 4.8 months) or overall survival (OS, 11.0 vs 10.3 months) or tumor response rate (31% vs 30%) compared to placebo group.

#### Radiotherapy

Reymen et al. investigated the potential of NTG as a radio-sensitizer on 42 patients with stage IB-IV NSCLC. A NTG patch (25 mg) was applied at least 2 h before the first radiation session of the day and removed only after the last session in case of bi-daily treatments. The clinical trial was stopped prematurely because the NTG did not improve overall survival (OS) and could not reduce tumor hypoxia in association with radiotherapy. The small sample size (42 patients) combined with the heterogeneity of patient characteristics and treatments modalities have complicated the analysis of the results in particular the overall survival in subgroup [94].

### Prostate cancer

A phase II clinical trial was completed in 2009 on prostate cancer by Siemens et al. [95]. A preclinical study indicated that NO plays a significant role in the hypoxia and in the development of the prostate cancer [77]. This clinical trial was realized on 29 men who experienced an increase in prostate-specific antigen (PSA) level after surgery or radiotherapy. Patients were treated continuously with slow-release NTG patches delivering a low-dose of NTG (0.033 mg/h) for 24 months. The primary evaluation criteria were PSA doubling time (PSADT). Before treatment, the PSADT was 13.3 months and after NTG treatment 31.8 months [95]. This clinical trial showed that the NTG can improve the PSADT by attenuating hypoxia-induced progression of prostate cancer. NTG has a beneficial antitumor effect in patients with relapsed prostate cancer.

### Liver cancer

In 2012, a randomized study was carried out to investigate potential benefit of NTG on the delivery and effectiveness Lipiodol (lymphographic agent) or Lipiodol/Doxorubicin emulsion after TAE (transcatheter arterial embolization) or TACE (transcatheter arterial (chemoembolization) respectively in patients with hepatocellular carcinoma (HCC). Lipiodol is selectively deposited in HCC tumors and used to visualize tumor-patient response by computerized tomography (CT). NTG improved the ability of Lipiodol to deposit in HCC tumors after TAE or TACE and GTN/TACE resulted in a greater reduction in tumor size compared to control groups [96]. Importantly, doxorubicin delivery may be enhanced because of the known NTG-mediated enhanced permeability and retention (EPR) effect [97].

### Rectal cancer

Recently, an open-label, nonrandomized, multicohort, dose escalation, phase I clinical trial was completed on 13 patients with locoregionally advanced operable rectal cancer [98]. The goal of this study was to evaluate the safety, the tolerability, the feasibility, the dose-limiting toxicity, and the maximum tolerated dose of NTG patch associated with 5-fluorouracil and radiation therapy. Three sequential doses of NTG were studied: 0.2, 0.4, and 0.6 mg/h (3 patients by dose). All patients received radiation therapy with continuous infusion of 5-fluorouracil per day for the duration of the radiation therapy. The NTG patch was applied 2 h before the radiation therapy and then during 12 h on days of radiation therapy (monday to friday). Overall, NTG patches were well tolerated by the patients and a complementary phase II clinical trial can be investigated with a dose of 0.6 mg/h of NTG [98].

The different association of therapeutic agents with NTG investigated in NSCLC, prostate, liver, and rectal cancer is summarized in Table 1.

**Table 1:** Summary table of clinical trials carried out on NTG in cancer therapy.

Stage (Patient number)	Associated treatment	Method of administeringNTG	Administration cycle	Ref.
**Non-small cell lung cancer (NSCLC)**				
**Phase II**				
Operable lung adenocarcinoma (17)	Surgery	NTG patch (25 mg)		[87]
Advanced lung adenocarcinoma (29)	Docetaxel (60 mg/m²) + Carboplatin (target area under the curve (AUC) 5)	NTG patch (25 mg)		[87]
IIIB/IV (120)	Vinorelbine (25 mg/m²) + Cisplatin (80 mg/m²)	NTG patch (25 mg)		[89]
IIIB/IV (66)	Vinorelbine (60–80 mg/m²) + Cisplatin (80 mg/m²)	NTG patch (25 mg)		[90]
IIIA-IIIB (35)	Vinorelbine (25 mg/m²) + Cisplatin (70 mg/m²) + radiotherapy	NTG patch (25 mg)		[91]
IV non-squamous (223)	Carboplatin (area under the curve 6) + Paclitaxel (200 mg/m²) + Bevacizumab (15 mg/kg)	NTG patch (15 mg)		[92]
III/IV (70)	Docetaxel (75 mg/m²) + Carboplatin	Micro-pump of NTG (20-50 µg/min)		[88]
IB-IV (42)	Radiotherapy	NTG patch (25 mg)	Patch is applied 2 h before the radiotherapy session and removed after	[94]
**Phase III**				
IIIB/ IV (372)	Carboplatin + Gemcitabine (79%) Or Carboplatin + Paclitaxel (18%) or Cisplatin + Vinorelbine (2%)	NTG patch (25 mg)		[99]
**Prostate cancer**				
**Phase II**				
Biochemical recurrence of prostate cancer after primary therapy (29)	Radiotherapy or surgery	NTG patch delivering 0.033 mg/h (1/6 of patch)	Patch applied continuously for 24 months	[95]
**Liver cancer**				
**Phase II**				
Hepatocellular Carcinoma (HCC) BCLC stage A or B (101)	Transcatheter Arterial (Chemo)Embolization (TAE/TACE)	Intravenous NTG (100 µg)	Injection of NTG via the catheter before the Lipiodol or Lipiodol/Doxorubicin emulsion	[96]
**Rectal cancer**				
**Phase I**				
Locoregionally advanced operable rectal cancer (13)	5-fluorouracil (225 mg/m²) + Radiotherapy	NTG patch delivering 0.2, 0.4 or 0.6 mg/h	Patch is applied 2 h before the chemoradiotherapy session and 12 h each day of chemoradiotherapy. The 5-fluorouracil is administrated with continuous infusion throughout duration of the radiotherapy	[98]

The arrow indicates the administration of chemotherapies or the surgery on day 1. In all clinical studies right above, the chemotherapies were injected on day 1 (as indicated by the arrow in Table 1) except for vinorelbine which is also injected on day 8. Different association of chemotherapies can be used for the treatment of NSCLC like docetaxel and carboplatin, vinorelbine and cisplatin or carboplatin and paclitaxel with bevacizumab (a monoclonal antibody directed against VEGF). The NTG patches were applied a few days before administration of the chemotherapy (3, 2 or 1 days) and then for 5 days.

*B* Bevacizumab, *Ca* Carboplatin, *Ci* Cisplatin, *D* Docetaxel, *G* Gemcitabine, *NTG* Nitroglycerin, *P* Paclitaxel, *V* Vinorelbine.

### Ongoing clinical trials

Three clinical trials intended to evaluate the antitumor potential of NTG in combination with other therapies have been conducted although the results not available yet (Table 2). The first study (NCT00616031) was a randomized phase II clinical trial realized by Yasuda et al. and investigated the effects of NTG in addition with carboplatin and paclitaxel for the treatment of previously untreated stage IIIB/IV NSCLC. This study would allow to verify if the poor results obtained by Dingemans et al. [92] are due to the interference of Bevacizumab with NTG or to the combination of chemotherapy used. The second study (NCT04338867) was a phase II clinical trial realized on 96 NSCLC patients with brain metastases. It investigated the effect of NTG combined with whole-brain radiation therapy (WBRT), the standard treatment for multiple brain metastases. The results were published recently but the article was temporally removed. In parallel, a phase III clinical trial (NCT01704274) was completed on 60 prostate cancer patients with biochemical recurrence after primary therapy (surgery or radiotherapy). This study seems to follow the first phase II trial published in 2009, demonstrating a beneficial effect of a low dose of NTG on PSADT in patients with biochemical recurrence after surgery or radiotherapy [95]. In this new study, the authors compared the efficacy of a low dose (0.0285 mg/h) and a high dose (0.057 mg/h) of NTG on the prostate cancer.

**Table 2:** Summary table of ongoing clinical trials carried out on NTG in cancer therapy.

Stage	Patient number	Associated treatment	Method of administeringNTG	ClinicalTrials.gov Identifier
**Non-small cell lung cancer (NSCLC)**				
**Phase II**				
Previously untreated stage IIIB/IV NSCLC	150	Paclitaxel and Carboplatin (6 cycles)	NTG patch	NCT00616031
NSCLC with brain metastases	96	Whole brain radiation therapy (WBRT)	NGT patch (36 mg from monday through friday throughout WBRT administration (10 days))	NCT04338867
**Prostate cancer**				
**Phase III**				
Biochemical recurrence of prostate cancer after primary therapy	60	Surgery or radiotherapy	NTG patch (0.0285 mg/h or 0.057 mg/h)	NCT01704274

## Conclusion

The putative repurposing of NTG in cancer therapy in order to overcome resistance to standard therapies is a recent strategy currently under investigation that aims to improve the management of cancer patients. A growing body of preclinical studies found clear evidence for the sensitizing effect of NTG on various anticancer therapies. Despite some significant outcomes in the field of clinical trials, much remains to be done to determine the best combination of therapies to use with NTG. How to combine NTG considering standard therapies, i.e., timing, frequency and for which type of cancer patient is still an open question. Better understanding the mode of action of NTG regarding its concentration, due to potential ambivalence of NO released, and effects within the temporal dynamics of TME would help to delineate the best responsive cancer patients most likely associated to a tumor-specific molecular signature.
